# Responding to historical injustices: Collective inheritance and the moral irrelevance of group identity

**DOI:** 10.1177/14748851221100094

**Published:** 2022-05-24

**Authors:** Santiago Truccone-Borgogno

**Affiliations:** Institute of Philosophy, 27267 University of Graz, Austria

**Keywords:** Indigenous people, group identity, Parfit, public evils, reparation, supersession thesis, Waldron

## Abstract

I argue that changes in the numerical identity of groups do not necessarily speak in favour of the supersession of some historical injustice. I contend that the correlativity between the perpetrator and the victim of injustices is not broken when the identity of groups changes. I develop this argument by considering indigenous people's claims in Argentina for the injustices suffered during the Conquest of the Desert. I argue that present claimants do not need to be part of the same entity whose members suffered injustices many years ago. For identifying the proper recipients of reparation, all that is necessary is that the group who suffered the historical injustice under consideration has survived into the present. I also support a view upon which present living members of a certain group have reasons to redress those injustices perpetrated by their predecessors if they are relevantly connected with each other. In particular, by relying on the notion of collective inheritance, I argue that if present-day members of a certain group claim that they are the continuation of the group whose past members bequeathed them certain goods, they cannot consistently reject such a membership when the very same people legated them certain evils.

## Introduction

The Argentine state has a long history of committing injustices against indigenous people. The most severe of many injustices was the *Conquest of the Desert*, which took place in the late 19th century. In the first stage from 1875 to 1878, a giant ditch was dug to prevent the indigenous population from crossing the frontier. The second and most famous stage began in 1878 and consisted of a set of aggressive military campaigns led by Julio Roca. This stage ended when Roca's troops reached *Choele-Choel* Island on 25 May 1879. The final stage ended with the surrender of the *Lonko* (Chief) Sayhueque in 1885. Among the consequences of the Conquest of the Desert are the following: Many lands previously occupied by indigenous people were appropriated by the past members of the Argentine state. Thousands of indigenous peoples were killed or profoundly harmed. Those who survived those military campaigns were confined to prisons or labour camps^1^ or were expelled to the southern zones of what today are the states of Argentina and Chile (Bustos-Videla, 1964: 44–56; Marimán-Quemado, 2006: 113).

One of the main difficulties for redressing historical injustices in the present is associated with changes in circumstances. From a 21st-century perspective, it is not always obvious that 19 th-century injustices still require reparation. Jeremy Waldron noted this problem with his supersession thesis which asserts, ‘Historic injustice may be overtaken by changes in circumstances so that a situation that was unjust when it was brought about may coincide with what justice requires at a later time’ (Waldron, 2004a: 237). Most discussion of the supersession thesis has focussed on how changes in circumstances that affect a particular distribution of goods might outweigh the reasons for redressing the effects of historical injustices (Lyons, 1981; Meisels, 2009: Ch.5; Sanderson, 2011; Waldron, 1992, 2004a, 2004b). In this paper, I will not consider how distributive justice concerns might supersede historical injustices. Instead, I will focus on a specific change in circumstance that is less explored in the literature – how changes in circumstances might affect the identity of the parties involved in past injustices.

Chandran Kukathas discusses this point and argues that ‘For the paying of reparations to be defensible, it must be possible to identify two kinds of agent: the victim of the injustice, to whom the reparation is owed, and the perpetrator or beneficiary of the injustice who can be held accountable for the wrong or liable for the cost of restitution’ (2006: 330). Requiring the identification of perpetrators and victims is relevant because the commission of some injustice generates a correlativity between them. This *correlativity requirement* lies at the core of all viable accounts of corrective justice. As Jules Coleman asserts, ‘In every account of corrective justice, there is presumed to be a relationship between the parties that make the claims of corrective justice appropriate to them – and not to others’ (1995: 66). This means that those who have suffered particular injustices have claims not against everyone but against someone in particular. The idea is that, after an act of injustice was committed, the claims for corrective justice are restricted to those parties involved in the commission of the injustice. The *correlativity requirement* limits the claims for reparation in the sense that those who suffered the consequences of historical injustices have claims for reparation not against everyone but only against those who perpetrated such injustice. Therefore, concerning the Conquest of the Desert, indigenous people's current corrective justice claims would be valid only as long as victims and perpetrators of the past injustice have survived into the present. Showing these points would not have been as problematic in the late 19th century in discussing appropriate responses to the Conquest of the Desert because both perpetrators and victims of the injustice would have been easier to identify. Nevertheless, more than 130 years have passed since the Conquest of the Desert, and all involved individuals are dead. The challenge is to explain how the correlativity between victims and perpetrators has endured even if no currently living person was alive when the Conquest of the Desert took place.

In response to this problem, many authors claim that even if those persons involved in past injustices are dead, such a fact is insufficient for showing that the injustice was superseded. This is because those groups who suffered past injustices might have endured into the present (Meisels 2009: 64–65; Spinner-Halev 2012: 63). Concerning those demands held in the name of some collective entity, these views often stipulate that for a claim of corrective justice to be justified, those pressing claims today must be part of the very same group or groups who suffered past injustices (Meisels 2009: 64–65; Waldron 2004b: 64). In this view, the correlativity between perpetrators and victims is maintained as long as the groups or communities involved in the past injustice have survived into the present. According to this account, in the case of Argentina, it must be shown that present living members of those groups making claims today are part of the same groups that suffered the Conquest of the Desert. However, the history of divisions and fusions among indigenous groups in Argentina has likely led to changes in their identity. Hence, the members of indigenous groups claiming reparation today would not be part of the same communities who suffered injustices in the past. Thus, on this account, it might be possible that those groups who suffered past injustices have not endured into the present. If this is the case, the *correlativity* between the perpetrators and the victims of the injustice has been broken and the past injustice has been superseded by changes in circumstances.^2^

This paper argues against this view. It contends that for a claim of corrective justice to be justified, claimants today do not need to be part of the *same* group whose members suffered injustices in the past. All that is necessary is that the groups who suffered historical injustices have endured into the present. As I argue, these are two different assertions that lead to different practical conclusions. If I can show this, then, the correlativity between the perpetrators and victims is not necessarily broken by changes in the identity of the parties involved in the original injustice.

Still, in the case of Argentina, even if it is accepted that those groups who were part of the Conquest of the Desert endured into the present, it is sometimes suggested that a vast majority of present-day non-indigenous Argentine citizens are not successors of those who perpetrated the Conquest of the Desert. A large portion of the Argentine population sees themselves as ‘*hijos de los barcos’* (descendants of the ships). They regard themselves as descendants of immigrants who arrived in the country after the military campaigns occurred. Hence, they believe they have no relationship with the perpetrators of the injustice and claim no responsibility for redressing the historical injustice committed against indigenous people during the Conquest of the Desert.

In response to this objection, I show why currently living non-indigenous Argentine citizens are not only successors of those who perpetrated the Conquest of the Desert, but also that they ought to be regarded as members of the *same* community. In particular, I argue that if non-indigenous Argentine citizens today claim that they themselves and those who bequeathed them certain goods are part of the same community, they cannot consistently reject such a membership when the very same people legated them certain evils.

## Numerical identity and the continued existence of groups over time

Considering the identities of the parties involved in some past injustice, Jeremy Waldron asserts that for defending that some past injustice has not been superseded, the entity who has suffered the injustice many years ago must have endured into the present (Waldron 2004b: 64). If this is the case, the correlativity between the parties involved in the injustice might not have been broken. Hence, present-day members of the group or community who suffered the injustice might have valid claims for reparation against the present-day members of the group or community who has perpetrated it.^3^

Waldron gives a specific example regarding the problem of defining a group's identity. He quotes a New Zealand case regarding Māori fishing rights expropriated by the 19th-century colonizers. Waldron notes that the Māori's fishing rights were stolen from the traditional rural tribes (*iwi*) so that their tribal way of living was no longer viable. As a consequence, there was a vast urban migration. Because the Māori are communal people, they formed urban groups to cover the functions historically performed by the tribe which eventually led to the creation of the *Urban Māori Authorities* (*UMA*). The Court of Appeal had to decide whether only the *iwi* or both the *iwi* and the *UMA* should be considered the relevant entity regarding the past violation of Māori fishing rights (Waldron, 2004b: 65–66). In this case, the Court of Appeal ruled that the fishing rights should be returned to the *iwi* but not to the *UMA*. The decision was based on the argument provided by the speaker of the Treaty of Waitangi Fisheries Commission claiming that ‘since the *iwi* had had the fishing rights wrongfully taken away from them, it is to *iwi* that they should be returned: *UMA* had not suffered comparable injustice, for *they did not exist at the time* the expropriation took place, and so they were not entitled to any redress’ (Waldron, 2004b: 66, *my emphasis*).

In that decision, the *identity* between the past Māori and the current *iwi* is the criterion used for identifying the entity whose claim is justified. However, *identity* is a term that has multiple fruitful uses. Thus, the first issue is to specify which kind of identity ought to be considered for assessing the survival or endurance of groups over time. Derek Parfit distinguishes between *numerical* and *qualitative* identity. He exemplifies this distinction by claiming that two white billiard balls are *qualitatively* identical. However, he asserts that if we take one of these balls and paint it red, the two balls are no longer *qualitatively* identical. Nevertheless, the current red ball is *numerically* identical to itself when it was white (Parfit, 1984: 201). From this perspective, *qualitative* identity is used for asserting that two different things share some relevant property that makes them equal. Instead, *numerical* identity is related to the fact that some entity has not disappeared or has not been destroyed even though some of its properties might have changed.

Imagine that the members of one group, *Perpetrator,* commit an injustice against the members of another group, *Victim*. Imagine too that there is a third group precisely like *Victim* called *Bad-Luck,* but with one difference. *Bad-Luck* has not suffered any injustice. In this situation, even if *Victim* and *Bad-Luck* are *qualitatively* identical, only *Victim* has justified claims for reparation against *Perpetrator*. This is because the injustice committed by *Perpetrator* against *Victim* generates a correlativity only between *Perpetrator* and *Victim*. *Qualitative* identity is not relevant for establishing such a relationship. Instead, what seems to be important when we think of claims for redressing historical injustices is *numerical* identity. This is because our primary concern refers to whether or not the entity that suffered injustices in the past has endured or survived into the present.

I argue that for the continuing existence of groups, numerical identity is not necessary either, but only *something* that is contained in the numerical identity of groups.^4^ Imagine a group G, which had a prominent chief, the *big chief,* and she has two twin children. The *big chief* has died, and group G's rules say that leadership must pass on to her heirs. When leadership passes on from the *big chief* to her heirs, the two future-chiefs-to-be are separated and the members of group G are divided into two groups. After the division, each group believes that it is G, has the memories of G, follows G's culture, institutions and traditions, and in everything else is like G.^5^

Does group G continue to exist after its division? This question has four possible responses: (1) G no longer exists; (2) G continues to exist as one of the two groups; (3) G continues to exist as the other of the two groups; (4) G continues to exist as both groups.^6^ The first possibility (1) is not a good answer. To see this, suppose that instead of G having been divided, half of the group has been destroyed. In this case, we would claim that the group G continues in existence. So, if group G endures when one half has been destroyed, it seems that G has to endure as well when that half has not been destroyed but merely separated. Therefore, response (1) – that is, group G no longer exists after its division – is false. If we accept this, possibilities (2) and (3) have to be false as well. If half of the group is enough for asserting the survival or endurance of the group and the two separated halves are relevantly similar, then it makes no sense to claim that group G continues to exist as only one of the resulting groups.

The last possibility is (4), that is, group G continues to exist as the two resulting groups. This might be explained, because the relation between G and the two resulting groups contains all the relevant elements that are contained in the ordinary survival of groups. I have claimed that group G endures if half of it is destroyed and the other half continues to exist. In this case, the relation between group G and the remaining group contains all the necessary elements for the endurance of the group over time. Therefore, if each half of the group, although separated, continues to exist, the relation between group G and each of the resulting groups has to contain all the necessary relevant elements in the ordinary endurance or survival of groups as well.^7^

The previous case shows that *numerical identity* does not matter or, at least, is not necessary for claiming that some group has survived or endured after its division. This is clear since numerical identity is a transitive relation, and the two resulting groups are not the same. Although it is plausible to assert that G is numerically identical to one of the other groups G_1_ and it is plausible to claim that G is numerically identical to the other group G_2_, it is not true that G_1_ and G_2_ are numerically identical to each other. However, even if the resulting groups are not numerically identical to each other, the relations between each of them and the original G contains all the vital elements required for the ordinary endurance of groups over time. If numerical identity were a necessary element for the continued existence of groups, we would have to claim that group G has not survived as either G_1_ or G_2_. However, this is not the case. Group G continues to exist as both groups G_1_ and G_2_. Thus, the maintenance of numerical identity is not required for the continued existence of groups over time.

The previous conclusion is important because, in the history of groups, there have been many divisions, and such divisions have usually led to the consequence that some of the resulting groups are not numerically identical to the groups who suffered injustices in the past. In this way, it is denied that in the present the *correlativity* between the perpetrators and the victims of some past injustice still exists. The New Zealand case in which the Court of Appeal denied that the *UMA* deserves reparation for the stolen fishing rights exemplifies this point. The Court denied the *UMA's* claims because, according to them, only the *iwi* but not the *UMA* existed at the time in which the expropriation of fishing rights took place (Waldron, 2004b: 66). In this case, the Court of Appeal seems to have taken *numerical identity* as the relevant criterion for claiming that the members of the *iwi* but not the *UMA* have justifiable claims to the benefits of the fishing rights. According to the criterion used in this decision, the correlativity generated in the past, when the government of New Zealand stole the Māori fishing rights, is maintained only concerning the *iwi* but not the *UMA*.

Taking numerical identity as the only relevant criterion for ascertaining the endurance of groups over time is a mistake. As I have analysed in the case of group G, in order to claim that some group continues to exist it is not necessary to prove that the current claimants are members of the group that is numerically identical to the group who suffered the past injustice. The only thing relevant for claiming that some group has endured over time is that the groups that exist today are in an appropriate relationship with the original group. This proper relationship does not need to entail the maintenance of the numerical identity. It could be that two different groups would be related to one old group in a similar way so that we might claim that the old group has endured as the two new groups. If we accept this, the correlativity generated between—for instance—the New Zealand government and the Māori might be maintained concerning both the *iwi* and the *UMA*. We do not need to choose between them. Both groups might be in an appropriate relationship with those who have suffered historical injustices.

## Divisions and fusions among indigenous peoples

If the argument outlined above is correct, it has significant consequences for redressing historical injustices perpetrated against indigenous peoples in Argentina during the Conquest of the Desert. *Mapuches* (also called *Araucanians), Tehuelches*, and *Ranqueles* are among the groups that suffered those injustices. Consider the case of the Mapuche people, which is the set of groups that is currently most numerous and most active in their claims for receiving reparation for the historical injustice their predecessors suffered.^8^ The issue is that after the Conquest of the Desert, different Mapuche communities were separated not only because the national borders of Argentina and Chile divide their ancestral territory (the *Wallmapu)* but also because Argentina is a federal state. Each of its provinces has its own legal system and, to some degree, they have different requirements for recognizing the formal existence of indigenous peoples. The disparity of legal requirements in different provinces contributed to changing Mapuche's numerical identity and way of living to different degrees. However, the maintenance of numerical identity is not necessary for claiming that some group has continued to exist over time. Hence, the fact that—nowadays—several Mapuche groups are not numerically identical to those who suffered past injustices does not speak against considering that the ‘old’ Mapuche groups as having endured into the present. That mere fact is not strong enough for breaking the *correlativity* between the perpetrator of the past injustice and the indigenous communities currently claiming reparation. Even if we might identify changes in the identity of groups, such variations do not imply that the groups or communities who suffered past injustices have ceased to exist. Therefore, changes in groups’ identity do not necessarily lead to the supersession of injustice.

There is a further point worth noting. The fact that the maintenance of the numerical identity of a group is not a necessary element for its continued existence over time works in the opposite direction as well. Like divisions, it is possible to imagine cases of *fusion.*^9^ Currently, in Argentina, many demands are not made on behalf of the Mapuche people but on behalf of the Mapuche–Tehuelche community.^10^ There is a well-known controversy both in Argentina and Chile about whether these communities were part of the same people in the past. Some scholars claim that the Tehuelches were part of the larger Mapuche community. They claim that the Mapuche are indigenous people who have lived on both sides of the Andes (*Füxa Mawiza)* since, at least, the year 660 AD and in a more organized way since the 16th century (Marimán-Quemado, 2006: 61). These scholars claim that the confusion regarding the possibility that the Mapuche and the Tehuelche may not have been part of the same people relies on the fact that the name *Mapuche* was given to the whole community many years after it was organized (Millalén-Paillal, 2006: 6–19). Therefore, according to this view, it makes no difference if the community presents itself as Mapuche or Mapuche-Tehuelche. In both cases, the current claimant would be the continuation of the old group or people who suffered injustices during the Conquest of the Desert.

However, other scholars have asserted that the Mapuche people originally belonged to Chile (Canals-Frau, 1946; Casamiquela, 1985). These authors claim that in southern Argentina, there has never been, at least as a *first-nation,* a Mapuche people. The Argentine indigenous people, according to them, were the Tehuelches. For example, Casamiquela asserted, ‘Mapuche is the name given to the people who lived and is currently living in the Araucania [Chilean territory]’ (1985: 9). These authors distinguished both communities because, according to them, Mapuche people were initially farmers, while Tehuelches were initially hunters (Casamiquela, 1985: 14). According to this line of thinking, that agricultural practice among southern indigenous peoples in Argentina occurred after it occurred in Chile shows a process of *Mapuchinization* (*Araucanization*) of the Argentine indigenous peoples.

This group of scholars disagree among themselves regarding the period in which the Chilean Mapuche (or Araucanians) arrived in Argentina. Those enrolled in the *historic-cultural* school of ethnology claim that the Araucanization of the pampas was a process that took place between the 16th and the early 18th century. A different group of scholars—enrolled in the *social-anthropology school—*asserts that the Araucanization of the pampas occurred later and ended in the 19th century.^11^ If we accept the first of these two positions, it makes no difference whether the Mapuche and the Tehuelche were initially part of the same group. It is irrelevant because the fusion of these two groups would have taken place long before the Conquest of the Desert.

Nevertheless, let us accept, for the sake of argument, that the position of those who claim that the Mapuche and the Tehuelche did not merge before the Conquest of the Desert is correct. Let us also accept that the Conquest of the Desert was a set of injustices committed against the Tehuelches and not against the Mapuche people. Further—as this narrative sometimes goes—let us assume that the Tehuelche and the Mapuche people fully merged in 1886, that is, a year after the Conquest of the Desert ended. Furthermore, let us assume—as this view also asserts—that the *pure* Tehuelche disappeared a couple of decades after the ‘last 200 Tehuelche’ were identified in 1967.^12^ What follows if we accept all these facts is that the members of the groups who are currently claiming reparation are not the same entity or groups who suffered injustices in the past. There is not a numerical identity between them. Thus, in this view, contemporary claimants are not members of the same group or groups who suffered the Conquest of the Desert. This suggests that the *correlativity* between the community who perpetrated the injustice and the group or groups who are currently claiming reparation has been broken.

However, fusions—like divisions—do not imply that the groups who merged ceased to exist. On Parfit, at least in regard to personal identity, it is possible to assert that the agent who is going to merge with another survives if two conditions are met. First, if there is a relevant connectedness between the old agents and the newly merged agent to an important degree. Second, if each of the old agents values the new merged agent's features (Parfit, 1984: 299). These two conditions can be applied to the situations of groups as well.

Let us start by analysing the first requirement. In the literature about the endurance of groups over time, relevant connectedness can be understood as what Tamar Meisels calls *cultural continuity* (Meisels, 2009: 64). If we focus on the Mapuche–Tehuelche situation, even if the process of fusion ended after the Conquest of the Desert, it is possible to see cultural continuity between the old groups and the new ones. Even under the assumption that the Mapuche and the Tehuelche were not initially part of the same people, it is sometimes recognized that before the so-called process of *Araucanization* (*Mapuchinization),* a situation of *Tehuelchization* took place, and many Mapuche groups adopted some customs that initially belonged to the Tehuelche (Aguerre, 2008: 27–28). For instance, groups of Mapuche and Tehuelche continued to live by hunting and grazing as the old Tehuelche did (Martinez-Sarasola, 1998: 115). Furthermore, these groups also incorporated certain agricultural practices that were held among the old ‘Chilean’ Mapuche (Casamiquela, 1985: 14). Similar patterns of fusion have taken place concerning their political organization, cultural customs (Canals-Frau, 1946: 162), religious practices, language and clothing (Mandrini and Ortelli, 1995: 141–146). This process of fusion was favoured by some of the policies the Argentine government pursued after the Conquest of the Desert since many Tehuelche and Mapuche survivors were forced to live together (Martinez-Sarasola, 1998: 178). Therefore, it seems that there was an important degree of connectedness between the new and the old groups, as the first condition of the endurance of groups requires.

However, what about the second requirement? How did the groups regard the fusion into new ones? This is a complicated question because it is hard to know what they thought about the new groups’ characteristics. Furthermore, the problem is that fusions are often accompanied by changes in culture, and ‘changes in culture pose a challenge to coherence and continuity’ (Føllesdal, 1996: 9). This, of course, does not mean that any change in culture and practices make groups no longer to exist. However, as Føllesdal asserts ‘cultural changes should not be too abrupt, since members of a culture have an interest in revising their plans as options and consequences change’ (1996: 9). For this reason, if one community had suddenly imposed certain practices on another, the case for the survival of the second would have been much more difficult to assert. Hence, when claiming that the old group continues to exist, it seems necessary that the members of the past group are able to adapt their life plans to new circumstances. Otherwise, it would be hard to explain how the new cultural, societal and institutional norms are their own.

So, how did the Mapuche and Tehuelche regard the fusion into a new group? What about the second requirement? The battles of *Senguer*, *Languiñeo* and *Shotel-Kaike*, which are among the most famous encounters between Tehuelches and Mapuches at the beginning of the 19th century seem to speak against considering both groups as having continued to exist after their fusion. However, over time, the relationship between different groups mutated, and links of marriage, filiation and commerce resulted in a new ethnicity (Aguerre, 2008: 44–46). It seems that members of both groups had certain and significant control over the direction and speed of change. In the end, in this narrative, the fusion between the Mapuche and the Tehuelche was a long process that included a complex network of mutual interchanges. Several practices were gradually adopted, and others were gradually dropped until a moment in which both groups were somehow indistinguishable from each other was reached (Mandrini and Ortelli, 1995). As some scholars assert, during this process, each of the entities adopted the practices of another that they considered more valuable up to the point of keeping only those that they valued more (Martinez-Sarasola, 1998: 115–116). This suggests that both groups saw specific value in the practices that, after the fusion, were incorporated into their ways of living, fulfilling the second condition for the survival of groups.

Suppose the last two criteria for assessing the endurance or survival of groups are accepted. In this case, the fact that the Conquest of the Desert was committed against the Tehuelche and not against the Mapuche does not mean that the initial correlativity between the perpetrator and the victims of the injustice was broken. Even according to this understanding of the historical facts surrounding the Conquest of the Desert, the Tehuelche did not cease to exist when they merged with the Mapuche. Therefore, the correlativity between the perpetrators of the injustice and the current Mapuche–Tehuelche people as the victim of such injustice can still be maintained.

## The continued existence of indigenous peoples into the present

So far, I have argued that although both cases of division and fusion speak against considering the group who suffered injustices in the past as numerically identical to the one (or those) who is (or are) claiming reparation in the present, neither of these situations necessarily speaks in favour of claiming that such a group has ceased to exist. Therefore, even if there were cases of fusion and divisions in the history of the Mapuche, Tehuelche, and other indigenous peoples, from that fact, it does not follow that the correlativity between the perpetrator of the injustices committed during the Conquest of the Desert and the current groups of indigenous claimants is broken. Nevertheless, I still have to explain what makes some groups continue to exist over time and give an account capable of explaining what it means that present-day members belong to a group that is in an appropriate relationship with the group that suffered past injustices.

As a starting point, we might claim that present-day members belong to a group that is in an appropriate relationship with the group that suffered past injustices when there is what Lukas Meyer calls *collective memory*, which ‘is to be understood not as a collection of individual memories, but rather as a socially articulated and socially maintained “reality of the past”’ (Meyer, 2001: 264). The notion of collective memory seems to be for groups what the continuity of memory is for individuals. As individual memory makes a person aware of their continued existence over time, collective memory seems to also make groups aware of their continued existence over time. In the case of the Mapuche people, this seems to be shown in their current narratives about the survival of *Mapudungun* (Mapuche language), storytelling like the *cycle of Elal* (Aguerre, 2008: 29–34), and through certain important practices that have roots, since at least the 19th century, such as the organization of the *Lof* (community) around the authority of a *Lonko* (chief), who is in charge of distributing, among other things, the usufructuary rights to the *Mapu* (territory) (Di-Giminiani, 2015: 492).

In my understanding, a *continuous collective memory* does not need to refer to socially articulated and socially maintained *direct* cultural, social and institutional connections between the two groups. Groups survive even under weaker conditions. It is not required that present-day members share some custom, practice, institution or memory with ancient members; the mere existence of a chain of such features is enough. In this understanding, a *continuous collective memory* should be interpreted as *overlapping chains* of socially articulated and socially maintained shared customs, practices, institutions and narratives. Therefore, groups do not cease to exist when they gradually changed their customs, institutions and social practices over time. If this condition is present, even if we cannot claim that the group is numerically identical to the one who has suffered historical injustices, we might rightly assert that we are addressing the correct group, i.e. the group that was, many years ago, in a correlative relationship with the perpetrators of the injustice. If the present-day groups have an appropriate relationship with the old one, the old group still exists, and the collective claims for the reparation of their members might be justified. This is because the required *correlativity* between victims and perpetrators, which stems from the past injustice, may still be in place.

## Collective inheritance and responsibility for redressing historical injustices

However, the claim that present-day members of indigenous people are the appropriate beneficiaries of reparations is less demanding than the claim that present-day non-indigenous Argentines have reasons of corrective justice for responding to those past injustices perpetrated by past members of the Argentine state. Concerning this second claim, additional support must be presented. To explain why currently living people have reasons of corrective justice for responding to historical injustices committed during the Conquest of the Desert, I will rely on an interpretation of Lukas Meyer's account of collective inheritance (Meyer 2001: 274–286; 1997: 147–150). Collective inheritance posits that certain goods can be collectively bequeathed to members of a specific ongoing society. Just as individual goods are handed down from past individuals to present individuals, other goods can be passed from all past members of a community to all present-day members of the community (Meyer, 1997: 147). For instance, in the case of Argentina, we might think about the legacies of the so-called *37 Generation* and the *80 Generation.* Many members of the *37 Generation* are thought of as the main creators of the Argentine Constitution of 1853. After some reforms, the Constitution is still valid today and incorporates among the most important legacies of the *37 Generation* ‘a division of powers, a system of checks and balances, a bill of rights, and also showed openness towards federalism’ (Gargarella, 2013: 34–35).

One of the most relevant legacies bequeathed by the *80 Generation* was their contribution to the long Argentine tradition of guaranteeing the principles of free and equal access to education.^13^ The tradition of ensuring free and equal access to education has roots in the Constitution of 1853. However, the *80 Generation* gave a considerable boost to the education system. For instance, during the Presidency of Roca, laws N° 1420 of 1884 and N° 1597 of 1885 (the so-called Avellaneda law in honour of its intellectual author) were enacted. The former ensured compulsory, free and secular elementary education, and the latter built the foundations of the modern Argentine university system. These ideas were brought forward in the *1918 University Reform of Córdoba*, whose result was a university system based on equal access, no tuition fees, academic freedom and co-government that includes faculties, students and graduates (Nino, 1992: 301).

These goods, that is, the Constitution, a system of public education and, in general, a certain social and institutional order, and civil liberties, can be considered what Joseph Raz calls public goods: ‘A good is a public good in a certain society if and only if the distribution of its benefits in that society is not subject to voluntary control by anyone other than each potential beneficiary controlling his share of the benefits’ (Raz, 1986: 198). Take, for instance, the public system of education. The Argentine system of education is a public good since no one can be excluded from enjoying the benefits of living in a society in which, for instance, the literacy rate is 99% of the population, and from a society that, according to the United Nations Human Development Report, has the highest position in Latin America in the Education index (its index is 0.842).^14^ Further, the possibility for a single individual to enjoy the benefits of living in a society with these features does not detract from the benefits enjoyed by others. It is an *inherited* public good because past generations wanted to bequeath the education system to all of us. The following quote by Domingo Sarmiento, the so-called father of the Argentine elementary education system illustrates this point clear:

An old dress covers the ragged man's nudity; but torn that dress, the nudity reappears, while the education of the ragged man, although slower in its effects, ends up providing the patient with the means to dress, and by breaking the thread of the tradition of the misery of the family in which he was born. Therefore, education is a capital put by present generations in the interest of future generations (Sarmiento, 1896: 34).^15^

One relevant consequence of having inherited public goods, such as a public education system, is that such an inheritance imposes certain duties on us. Meyer explains this point eloquently when he discusses some of the duties that stem from receiving a highly advanced system of tertiary education: ‘the respect owed to our predecessors’ sacrifices and savings, which were intended to benefit not only us but more remote future people as well, amounts to a general obligation not to dispose of or use up those goods for their own private interest’ (Meyer, 1997: 148). Consider *La Noche de los Bastones Largos* (The Night of the Long Batons), which is perhaps the most representative example of an attempt to dispose of a public good bequeathed by our predecessors. With the intention of dismantling the Argentine university system based on academic freedom, on 29 July 1966, during the dictatorship of Juan Carlos Onganía, the Argentine Federal Police arrested many students and professors of the University of Buenos Aires that opposed the dictatorship. As Carlos Nino recounts: ‘The universities were purged of dissidents; opponents were detained under a state of siege; and the government, inspired by right-wing sectors of the church, established strict standards of private morality’ (Nino, 1996: 50). The attacks on professors and students not only wronged them but also affected a public good that past generations intended to bequeath to the subsequent. Ongania's intervention caused mass resignations of professors and more than 300 emigrated. Further, many research teams and institutes were dismantled, and all public universities were deprived of their autonomy (Torre and De-Riz, 1993: 300).

We can use the concept of inherited public goods to explain some aspects of the wrongness of the actions performed during Ongania's dictatorship, even if it cannot be claimed that future people will have a right to a good system of education. This is because respect is owed to the sacrifices made by past generations in passing those goods on to the latter (Meyer, 1997: 148). Further, as Meyer asserts, ‘Typically, we inherit public goods, not as sole beneficiaries but as persons able to share and pass on these goods to our descendants’ (2001: 280). Therefore, in the case of Argentina, given that the system of education was likely to be bequeathed by the *80 Generation* with the intention to benefit future generations, the destruction of such a good generally implies the non-fulfillment of duties with respect to future generations.

Nothing I have said implies that present generations cannot deny or repudiate collective inheritances. In the same way that we might inherit public goods from past generations, we can also collectively inherit *public evils* (Meyer, 1997: 150). According to Meyer, an important feature of public evils is that ‘The harms that accrue to members of a minority from living under a regime that discriminates against them owing to their race, language, ethnicity or customs are not subject to voluntary control by these people’ (Meyer, 2001: 277). This feature can be exemplified when we look at the consequence of the Conquest of the Desert. Thousands of indigenous persons were killed or harmed, expelled to Chile or the southern zones of Argentina. Their lands were taken from them, and Argentine institutional order was imposed over them. Further, even among those indigenous people who did not suffer such extreme consequences, it was common to send them to factories and companies in the north of the country to be used as cheap labour. The rationale for this policy was that indigenous people would be quickly transformed and able to adopt ‘valid criteria of civilization’ (Levaggi, 1990: 453).

The injustices committed during the Conquest of the Desert were possible, in part, because there was a web of moral, legal and ideological ways of thinking that might still be present nowadays, even if there are no longer military campaigns against the indigenous population.^16^ These evils collectively inherited by Argentines today are public in the sense that ‘all members of the society tend to be negatively affected by the bad collective inheritance without normally having much control over the ways in which the degree to which they are going to be affected by this inheritance’ (Meyer, 2001: 278). In Argentina, this can be appreciated if we consider that while survivors of the Conquest of the Desert were incorporated into the larger society they were relegated to the poorest and lower segments, and they remain there (Svampa, 2016: 45). Furthermore, other signs for thinking that the legacies of the Conquest of the Desert can be gleaned from the 2012 Report of the Special Rapporteur on the Rights of Indigenous Peoples on the situation of indigenous peoples in Argentina:

The situation of indigenous peoples in the country with regard to land tenure is a result of the fact that historically they have been dispossessed of large tracts of their land by ranchers and by the operations of farming, oil, and mining companies on lands claimed by indigenous communities. The majority of indigenous communities in the country have not received legal recognition of their lands in line with their traditional ways of using and occupying those lands (Anaya, 2012: 7).

If it is true that past generations bequeathed these public evils to present generations, as seems to be the case, the next question is: What ought to be done with them? The answer: Given that these inherited evils have a negative impact on present and future people, as Meyer asserts, ‘we have good reasons for wanting—and may well be under an obligation—to dispose of the public evils we inherit’ (Meyer, 2001: 282). If we consider it valuable to live in a just society, or at least in one in which people can pursue autonomous lives, we have the duty to dispose of those public evils that may prevent both the establishment of such a society and that inhibit certain segments of the population from pursing their lives’ plans. Consider again the Conquest of the Desert and its lasting impact on currently living people. It follows from the arguments provided above that present-day Argentines have good reasons for disposing of the public evils they have inherited from the past.

This responsibility is not based on being members of the same community who committed past injustices. Rather, the responsibility for redressing those injustices is grounded on the collective inheritance received by our predecessors. However, one might deny this responsibility by rejecting the membership in the transgenerational community whose past members bequeathed those public evils to us. As Schaap has highlighted ‘as such, recognition of historical obligations does not depend on our identification with the people of the past’ (Schaap, 2005: 121). However, taking responsibility for historical injustices ‘obliges [present people] to see themselves as participants in a transgenerational relationship in which each generation inherits obligations from its predecessors and passes on obligations to its successors’ (Thompson, 2002: 18; also Schaap, 2005: 121).^17^ Given this, one might avoid or reject collective inheritances by denying the membership in a transgenerational community whose past members bequeathed these public evils and goods to the present members (Meyer, 2001: 283–284). Indeed, in the case of Argentina, some people claim that present-day non-indigenous Argentines have nothing to do with those injustices committed in the late 19th century. These people sometimes perceive themselves as descendants of immigrants that arrived later. As highlighted in the introduction of this paper, it is a common phrase among non-indigenous Argentines to claim that they descend from the *ships.*^18^ The idea refers to the massive wave of immigration received by Argentina in the late 19th and early 20th centuries. In 1869, before the Conquest of the Desert, Argentina's population was 1,830,214. By 1914, the population had more than quadrupled to 7,885,237 (Gallo, 1993: 84). Between the end of the Conquest of the Desert and 1914, Argentina received 5,185,288 immigrants (Cortés-Conde, 1993: 56). In 1914, around 33% of the Argentine population was foreign-born and another 25% were descendants of immigrants (Rock, 1993: 113). In light of these demographic data, the idea that many present-day Argentines have nothing to do with the members of the community that committed injustices in the late 19th century is plausible. As explained by Bashir Bashir, taking responsibility for historical injustices seems to become especially problematic ‘when the society is composed of successive and culturally diverse waves of immigration’ (Bashir, 2012: 134).

However, in the case of Argentina, there are at least two problems with rejecting such membership in the community that committed the Conquest of the Desert. First, being a member of a particular society is not something that people generally choose. Present-day Argentines are connected with past members, in part, by virtue of sharing a *continuous collective memory.* Continuous collective memory, it is worth recalling, is to be understood as a chain of overlapping socially articulated and socially maintained shared customs, practices, narratives and institutions. Based on the historical facts highlighted above, we can see how there is a continuous collective memory in the sense of the existence of a socially articulated narrative concerning, for instance, the origin of the Argentine education system. In this regard, no one denies, and as far as I know, in Argentine history, no one has denied the important work performed by Domingo Sarmiento and the *80 Generation*.^19^ There is, in Argentina, a clear continuous collective narrative about how the public education system was established in the 19th century. Furthermore, there is a collective narrative in the history of the creation and evolution of the modern state of Argentina that views indigenous peoples as ‘internal outsiders’ not included in the Argentine *us.* With roots in Domingo Sarmiento's dichotomy between *civilization* and *barbarism* and the ideas of the *80 Generation*, this narrative favoured the denial of the presence of indigenous people as members of Argentine society (Svampa, 2016). Indeed, such an exclusion has been considered ‘natural’ by many generations of Argentine citizens educated under the model of cultural uniformity for more than a century (Salgado, 2010: 150). Therefore, there seems to be a continued collective memory between present-day non-indigenous members of Argentina and those who belonged to the *80 Generation*, the generation that legated both the collective public good of a free system of education and the collective public evil associated with the lasting impacts of the Conquest of the Desert.

Second, there is a problem of consistency. Suppose people today reject the duties that stem from having inherited a public evil from past generations because they consider themselves members of a different community. In that case, they cannot consistently assert that they are part of the same community when the very same people bequeathed them a public good that is a constitutive part of the same collective public inheritance. In other words, present-day non-indigenous Argentines cannot simultaneously assert that they are and are not part of the same community as the members of the *80 Generation*. If non-indigenous Argentines today claim that they and those who bequeathed them a system of public education are part of the same community, given that the same generation that laid the foundation for such a system is the same generation that pursued the Conquest of the Desert, and further given that the public good and the public evil are part of the same collective inheritance, then present-day non-indigenous Argentines must also accept that they are part of the community that perpetrated that injustice. As such they have reasons for responding to its lasting impacts.^20^

## Conclusion

In this paper, I argued that current living claimants do not need to be part of the same group whose members suffered injustices many years ago. For identifying the proper recipients of reparation, all that is necessary is that the group who suffered the historical injustice continues to exist in the present. It requires a continuous collective memory between the members of the group who make claims today and those of the group who suffered past injustices. However, the existence of a continuous collective memory in no way presupposes group numerical identity. In other words, although the claimants for collective demands for reparation have to be members of groups that, having suffered historical injustices, have survived or endured into the present, it is not true that they have to belong to the very same groups who suffered past injustices. Thus, concerning those claims made in the name of the collective entity, the correlativity between the perpetrators and victims is not necessarily broken by changes in the identity of the groups involved in the original injustice. Therefore, if reparations are due, the group whose members share a continuous collective memory with past victims of the injustice are those who should receive reparation.

With respect to the responsibility for redressing historical injustices, I argued that if people today claim to be members of a community who bequeathed them public goods, these people cannot consistently reject such a membership when the very same people bequeathed them public evils as part of the same collective inheritance. In particular, I argued that since the *80 Generation* was responsible for the Conquest of the Desert, that currently living non-indigenous Argentine citizens are part of the same community as the members of the *80 Generation*, and that the lasting impacts of such injustice can be understood as an inherited collective evil, then currently living non-indigenous Argentine citizens have reasons for disposing of the inherited public evil associated with the lasting impacts of the Conquest of the Desert.

## Notes

1. Some *Lonkos* were kept captives in museums in Buenos Aires, Mar del Plata and La Plata (Marimán-Quemado 2006: 113).

2. This difficulty is one reason some authors focus on the continuity of structures rather than on the continuity of agents, and abandon the interactional approach in favour of the structural one. *See* Lu (2017: 159–166).

3. Claims for restitution and compensation are usually distinguished in the corrective justice literature. Restitution is often discussed with respect to territorial claims or cultural objects, and it requires returning the thing initially taken. Instead, compensation refers to those measures which aim to offset the consequences of injustices. Compensation might be demanded either because restitution is not possible or in addition to the restitution of goods, as a way of offsetting further consequences associated with the loss of the object or other types of injustices. On this distinction, *see* Meisels (2009: 53–58), Ivison (2006: 64). Since the correlativity requirement applies to corrective justice claims to both restitution and compensation, I will use ‘reparations’ as an encompassing term that includes both types of claims. Thank you to an anonymous reviewer for helping me to clarify this point.

4. My view is grounded on Derek Parfit's argument about personal identity, which, as I will show, can be rightfully transferred to the issue of the identity of groups, at least for asserting that some group has not ceased to exist. For the argument on personal identity *see* Parfit (1984: 253–266).

5. This case is analogous to Parfit's example about the unimportance of personal identity (Parfit, 1984: 253–255).

6. For the analogous set of responses concerning personal identity and the corresponding analysis, *see* Parfit (1984: 256–261).

7. For the analogous analysis of (4) concerning personal identity, *see* Parfit (1984: 262).

8. According to the last national population census, in 2010, 205,009 persons recognized themselves as Mapuche or descendants of Mapuche people. Mapuche people are 21.5% of the indigenous population in Argentina (INDEC 2012: 281).

9. This might be what Tamar Meisels has in mind when she claims in reference to contemporary corrective justice claims for historical injustices that ‘even if that harm was inflicted on their ancestors as members of distinct sub-groups of that culture it still seems plausible to view their descendants, contemporary group members, as the cultural heirs of that injustice’ (Meisels, 2009: 64). However, she does not fully explain how to deal with cases of fusions and divisions in detail.

10. As an anonymous review suggested, it is also possible to imagine cases of fusion between indigenous and non-indigenous persons (see Kukathas, 2006: 330–341). This is a possibility worthy of assessment. However, it is not part of the discussion about how to respond to injustices like the Conquest of the Desert in Argentina. The main point of contention relies on whether current claimants are members of the same group who suffered historical injustices or if they belong to different indigenous peoples. For this reason, I leave the analysis of this issue for another opportunity.

11. *See* Mandrini and Ortelli (1995).

12. *See* Ramos and Delrio (2005: 76).

13. Presidents Julio Roca, Nicolás Avellaneda and Domingo Sarmiento were among the most prominent members of the *80 Generation*. These three were also heavily involved in both the design and execution of the Conquest of the Desert and in the creation and implementation of the public system of education in Argentina.

14. See http://uis.unesco.org/en/country/ar?theme=education-and-literacy and http://hdr.undp.org/en/data (Accessed, 29 July 2020).

15. The translation is of my own.

16. However, it is undoubtedly true that indigenous people continue to be criminalized during specific demonstrations as was highlighted by James Anaya's report (2012: 13).

17. Thank you to an anonymous reviewer for pressing on this point.

18. For instance, when the Prime Minister of Spain visited Argentina in 2021, President Alberto Fernandez asserted, ‘Mexicans came from the Indians, Brazilians came out of the jungle, but we Argentines came from boats from Europe’. Similarly, in the World Economic Forum of Davos in 2018, former President Mauricio Macri asserted, ‘this is a natural association because in South America we are all descendants from Europe’.

19. In Argentina, Sarmiento symbolizes the image of a ‘good educator’ given his efforts in building a strong system of education (Gargarella, 2010: 170).

20. It is also the case that, arguably, the Conquest of the Desert benefited currently living non-indigenous members of Argentina. Thus, as current beneficiaries of such injustice, they might be remedially responsible for the harms currently suffered by indigenous people. *See* Miller (2007: 98–103).
